# *In Vitro* Metabolic Pathways of the New Anti-Diabetic Drug Evogliptin in Human Liver Preparations

**DOI:** 10.3390/molecules201219808

**Published:** 2015-12-04

**Authors:** Hyeon-Uk Jeong, Ju-Hyun Kim, Dae Young Lee, Hyun Joo Shim, Hye Suk Lee

**Affiliations:** 1 Drug Metabolism and Bioanalysis Laboratory, College of Pharmacy, The Catholic University of Korea, Bucheon 420-743, Korea; wjd1375@hanmail.net (H.-U.J.); jhyunkim@catholic.ac.kr (J.-H.K.); 2 Research Center, Dong-A ST Co., Yongin 446-905, Korea; dylee@donga.co.kr (D.Y.L.); shimhj@donga.co.kr (H.J.S.)

**Keywords:** evogliptin metabolism, human hepatocytes, cytochrome P450, UDP-glucuronosyl- transferases

## Abstract

Evogliptin ((*R*)-4-((*R*)-3-amino-4-(2,4,5-trifluorophenyl)butanoyl)-3-(*tert*-butoxymethyl)-piperazin-2-one), is a new dipeptidyl peptidase IV inhibitor used for the treatment of type II diabetes mellitus. The *in vitro* metabolic pathways of evogliptin were identified in human hepatocytes, liver microsomes, and liver S9 fractions using liquid chromatography-Orbitrap mass spectrometry (LC-HRMS). Five metabolites of evogliptin-4-oxoevogliptin (M1), 4(*S*)-hydroxyevogliptin (M2), 4(*R*)-hydroxyevogliptin (M3), 4(*S*)-hydroxyevogliptin glucuronide (M4), and evogliptin *N*-sulfate (M5)—were identified in human liver preparations by comparison with authentic standards. We characterized the cytochrome P450 (CYP) enzymes responsible for evogliptin hydroxylation to 4(*S*)-hydroxyevogliptin (M2) and 4(*R*)-hydroxyevogliptin (M3) and the UGT enzymes responsible for glucuronidation of 4(*S*)-hydroxyevogliptin (M2) to 4(*S*)-hydroxy-evogliptin glucuronide (M4). CYP3A4/5 played the major role in the hydroxylation of evogliptin to 4(*S*)-hydroxyevogliptin (M2) and 4(*R*)-hydroxyevogliptin (M3). Glucuronidation of 4(*S*)-hydroxy-evogliptin (M2) to 4(*S*)-hydroxyevogliptin glucuronide (M4) was catalyzed by the enzymes UGT2B4 and UGT2B7. These results suggest that the interindividual variability in the metabolism of evogliptin in humans is a result of the genetic polymorphism of the CYP and UGT enzymes responsible for evogliptin metabolism.

## 1. Introduction

Type II diabetes mellitus (DM) is a chronic metabolic disorder, characterized by relative insulin deficiency due to disorders of insulin secretion and insulin resistance, the prevalence of which has increased continually in the majority of countries [1]. Various classes of oral antidiabetic drugs can be used to control the blood glucose level and to prevent diabetic complications such as diabetic nephropathy and retinopathy [1,2].

Dipeptidyl peptidase IV (DPP-IV) inhibitors reduce the blood glucose level by inhibiting DPP-IV, a ubiquitous enzyme which rapidly degrades glucagon-like peptide 1 and glucose-dependent insulinotropic polypeptide, and many DPP-IV inhibitors—including alogliptin, anagliptin, gemigliptin, linagliptin, saxagliptin, sitagliptin, teneligliptin, and vildagliptin—have been developed as oral antihyperglycemic agents for the treatment of type II DM [1,2,3,4,5].

Evogliptin (DA-1229, trade name: Sugarnon^®^), a new, potent, and selective DPP-IV inhibitor [6,7,8,9,10,11], was approved by the Ministry of Food and Drug Safety of Korea as an oral antihyperglycemic drug for the treatment of type II DM on October 2 2015. Although the pharmacokinetic properties of evogliptin in humans have been reported [9,10], there is no report of its *in vitro* metabolism in humans. Metabolite identification and characterization of drug-metabolizing enzymes—such as cytochrome P450 (CYP) and UDP-glucuronosyltransferase (UGT)—responsible for the metabolism of drugs can facilitate prediction of interindividual variations in drug metabolism and pharmacokinetics, together with drug–drug interactions [12,13,14]. The purposes of the present study were to identify the metabolites of evogliptin formed after incubation with human hepatocytes, liver microsomes, and liver S9 fractions in the presence of cofactors using liquid chromatography-Orbitrap mass spectrometry (LC-HRMS), and to characterize the CYP and UGT enzymes responsible for evogliptin metabolism.

## 2. Results and Discussion

### 2.1. In Vitro Metabolic Profiles of Evogliptin in Human Hepatocytes, Liver Microsomes, and Liver S9 Fractions

**Figure 1:**
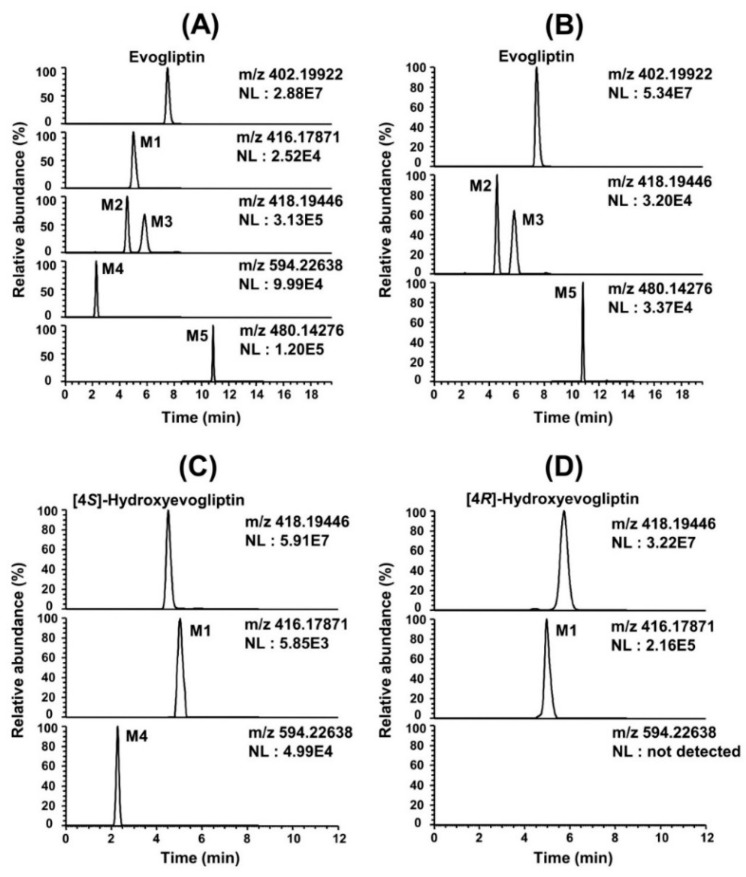
(**A**) Extracted ion chromatograms (EIC) of evogliptin and possible metabolites after incubation of evogliptin with human hepatocytes; (**B**) EIC of evogliptin and possible metabolites after incubation of evogliptin with pooled human liver S9 fractions in the presence of NADPH and PAPS; (**C**) EIC of 4(*S*)-hydroxyevogliptin and possible metabolites after incubation of 4(*S*)-hydroxyevogliptin (M2) with pooled human liver microsomes in the presence of NADPH and UDPGA; and (**D**) EIC of 4(*R*)-hydroxyevogliptin and possible metabolites after incubation of 4(*R*)-hydroxyevogliptin (M3) with pooled human liver microsomes in the presence of NADPH and UDPGA.

LC-HRMS analysis following incubation of evogliptin with human hepatocytes resulted in the formation of five evogliptin metabolites M1–M5 (Figure 1A). Following incubation of evogliptin with liver S9 fractions in the presence of NADPH and PAPS, three metabolites—M2, M3 and M5—were identified by LC-HRMS (Figure 1B). The retention time (t_R_) and accurate mass of protonated or deprotonated molecular ions ([M + H]^+^ or [M − H]^−^) and the characteristic fragment ions for evogliptin and five metabolites (M1–M5) are summarized in Table 1.

**Table 1:** Retention time and exact mass of the molecular ion of evogliptin and five metabolites identified after incubation of evogliptin with human hepatocytes, liver microsomes, and S9 fractions.

No.	Compound Name	Formula	Electrospray Ionization Mode	Detected Exact Mass (*m*/*z*)	Error (ppm)	Retention Time (min)
	Evogliptin	C_19_H_26_F_3_N_3_O_3_	Positive	402.19922	−1.7	7.51
M1	4-Oxoevogliptin	C_19_H_24_F_3_N_3_O_4_	Positive	416.17871	−1.1	4.97
M2	4(*S*)-Hydroxyevogliptin	C_19_H_26_F_3_N_3_O_4_	Positive	418.19446	−0.8	4.55
M3	4(*R*)-Hydroxyevogliptin	C_19_H_26_F_3_N_3_O_4_	Positive	418.19446	−0.8	5.83
M4	4(*S*)-Hydroxyevogliptin glucuronide	C_25_H_34_F_3_N_3_O_10_	Positive	594.22638	−0.9	2.27
M5	Evogliptin *N*-sulfate	C_19_H_26_F_3_N_3_SO_6_	Negative	480.14276	1.3	10.85

**Figure 2:**
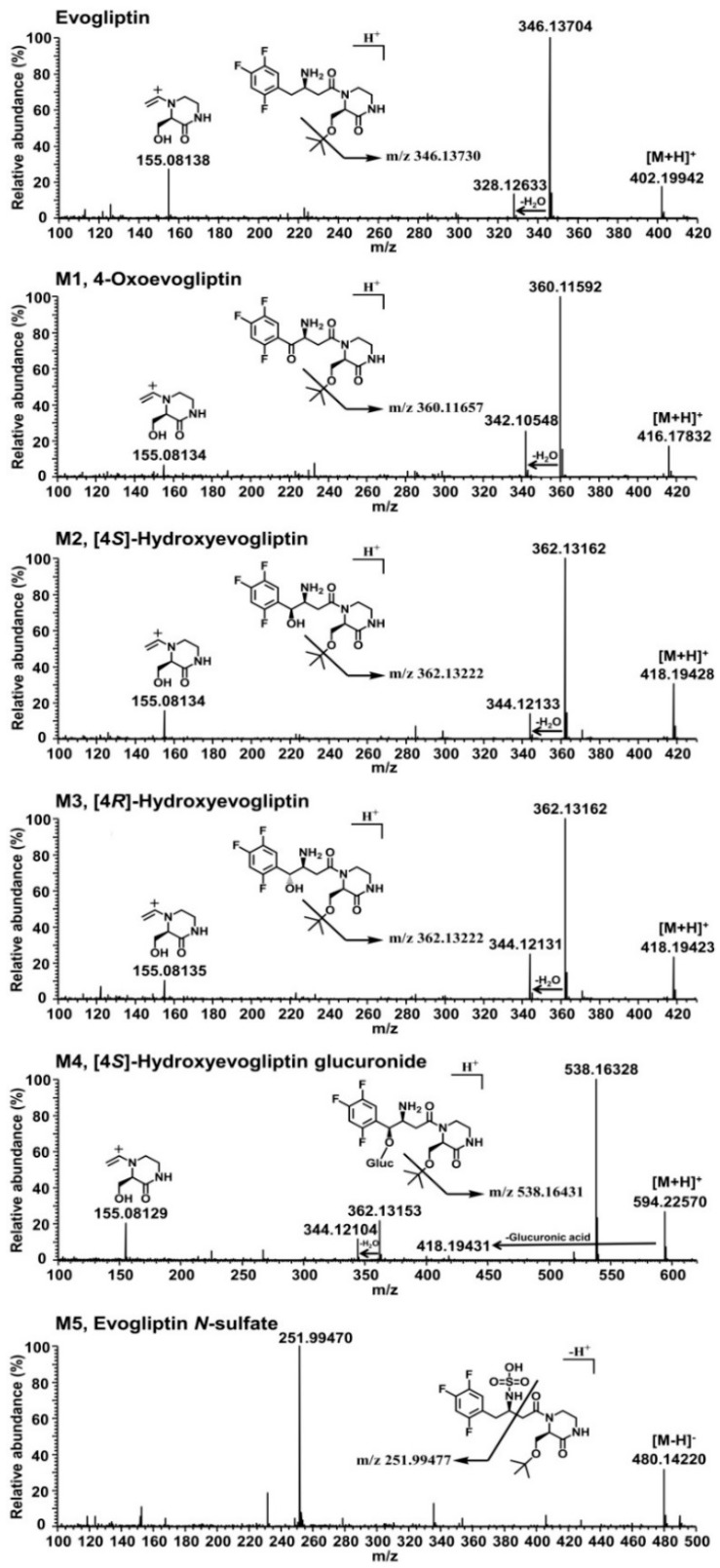
Product scan spectra of evogliptin and the five metabolites M1, M2, M3, M4, and M5 formed after incubation of evogliptin with human hepatocytes.

Five metabolites, M1–M5, were identified by comparison with the retention time and fragment ions of the corresponding authentic standards. The product scan spectrum of evogliptin showed a [M + H]^+^ ion at *m*/*z* 402.19942 with characteristic fragment ions at *m*/*z* 346.13704 (loss of a *tert*-butyl group from the [M + H]^+^ ion), *m*/*z* 328.12633 (loss of water from *m*/*z* 346.13704), and *m*/*z* 155.08138 (loss of 2-(2,4,5-trifluorophenyl)ethanamine from *m*/*z* 328.12633) (Figure 2).

M1 showed the [M + H]^+^ ion at *m*/*z* 416.17832, which was 14 amu more than the [M + H]^+^ ion of evogliptin, and yielded the product ions at *m*/*z* 360.11592 (loss of a *tert*-butyl group from the [M + H]^+^ ion), *m*/*z* 342.10548 (loss of water from *m*/*z* 360.11592), and *m*/*z* 155.08134 (loss of 2-(2,4,5-trifluorophenyl)ethanamine from *m*/*z* 342.10548) (Figure 2). Incubation of 4(*S*)-hydroxyevogliptin (M2) and 4(*R*)-hydroxyevogliptin (M3) with human liver microsomes in the presence of NADPH resulted in the formation of M1 (Figure 1C,D), suggesting that M1 was formed by 4-hydroxylation of evogliptin followed by dehydrogenation. M1 was identified as 4-oxoevogliptin by comparison with the retention time and MS/MS spectrum of the corresponding authentic standard. The peak area of 4-oxoevogliptin (M1) formed from 4(*R*)-hydroxyevogliptin (M3) was 40-fold higher than that of M1 formed from 4(*S*)-hydroxyevogliptin (M2) (Figure 1C,D), indicating that 4-oxoevogliptin (M1) was formed mainly from 4(*R*)-hydroxyevogliptin (M3).

M2 and M3 showed a [M + H]^+^ ion at *m*/*z* 418.19428, which is 16 amu more than [M + H]^+^ ion of evogliptin, indicating hydroxylation of evogliptin. The product scan spectra of M2 and M3 showed fragment ions at *m*/*z* 362.13162 (loss of a *tert*-butyl group from the [M + H]^+^ ion), *m*/*z* 344.12133 (loss of water from *m*/*z* 362.13162), and *m*/*z* 155.08134 (loss of 2-(2,4,5-trifluorophenyl)ethanamine from *m*/*z* 344.12133) (Figure 2). M2 and M3 were identified as 4(*S*)-hydroxyevogliptin (M2) and 4(*R*)-hydroxyevogliptin (M3), respectively, by comparison with the retention time and MS/MS spectra of the corresponding authentic standards.

M4 showed a [M + H]^+^ ion at *m*/*z* 594.22570, which is 176 amu higher than the [M + H]^+^ ion of 4-hydroxyevogliptin, indicating glucuronidation of 4-hydroxyevogliptin. M4 generated characteristic product ions at *m*/*z* 418.19431 (loss of glucuronic acid moiety from the [M + H]^+^ ion), *m*/*z* 362.13153 (loss of a *tert*-butyl group from *m*/*z* 418.19431), *m*/*z* 538.16328 (loss of a *tert*-butyl group from the [M + H]^+^ ion), and *m*/*z* 155.08129 (loss of 2-(2,4,5-trifluorophenyl)ethanamine and water from *m*/*z* 362.13153) (Figure 2). Human liver microsomal incubation of 4(*S*)-hydroxyevogliptin (M2) in the presence of UDPGA and NADPH resulted in the formation of M4 (Figure 1C). M4 was identified as 4(*S*)-hydroxyevogliptin glucuronide by comparison with the retention time and MS/MS spectrum of the corresponding authentic standard.

M5 showed a [M − H]^−^ ion at *m*/*z* 480.14220, which was 80 amu higher than the [M − H]^−^ ion of evogliptin, indicating sulfation of evogliptin. Since the [M + H]^+^ ion of M5 at *m*/*z* 482.15646 showed 195-fold lower peak intensity than that of the [M − H]^−^ ion of M5, we performed the product scan in negative mode. M5 generated the product ion at *m*/*z* 251.99477 [(2-(2,4,5-trifluorophenyl)ethylidene) sulfamate ion] (Figure 2). M5 was identified as evogliptin *N*-sulfate by comparison with the retention time and MS/MS spectrum of the authentic standard. *N*-sulfation of another DPP-IV inhibitor, sitagliptin, in human support that sulfation of evogliptin to M5 in human hepatocytes and liver S9 fractions [15].

**Scheme 1:**
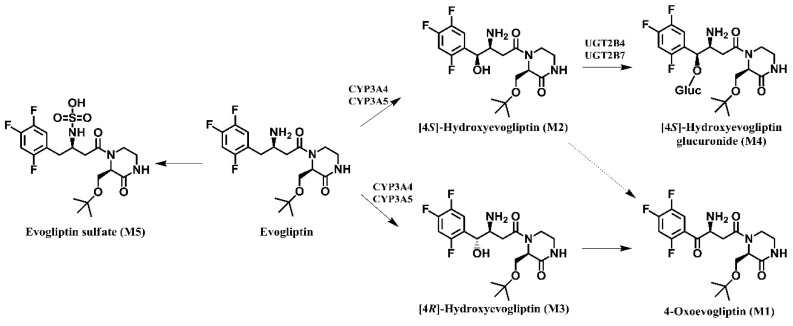
Possible metabolic pathways of evogliptin in human hepatocytes, liver microsomes and liver S9 fractions.

On the basis of these results, the possible *in vitro* metabolic pathways of evogliptin in human liver preparations are shown in Scheme 1: evogliptin is metabolized to 4-oxoevogliptin (M1), 4(*S*)-hydroxyevogliptin (M2), 4(*R*)-hydroxyevogliptin (M3), 4(*S*)-hydroxyevogliptin glucuronide (M4), and evogliptin *N*-sulfate (M5).

### 2.2. Characterization of Human CYP Enzymes Responsible for the Formation of 4(S)-Hydroxyevogliptin (M2) and 4(R)-Hydroxyevogliptin (M3) from Evogliptin

Screening for the metabolism of evogliptin at 5 and 50 μM to 4(*S*)-hydroxyevogliptin (M2) and 4(*R*)-hydroxyevogliptin (M3) using major human cDNA-expressed CYPs 1A1, 1A2, 2A6, 2B6, 2C8, 2C9, 2C19, 2D6, 2E1, 2J2, 3A4, and 3A5 isoforms showed that CYP3A4 played a major role in the formation of 4(*S*)-hydroxyevogliptin (M2) and 4(*R*)-hydroxyevogliptin (M3), with a minor contribution by CYP3A5 (Table 2).

**Table 2:** Formation rates of 4(*S*)-hydroxyevogliptin (M2) and 4(*R*)-hydroxyevogliptin (M3) from 5 and 50 μM evogliptin in human cDNA-expressed CYPs (*n* = 3, mean ± SD).

Human CYPs	4(*S*)-Hydroxyevogliptin (M2) (pmol/pmol CYP/min)		4(*R*)-Hydroxyevogliptin (M3) (pmol/pmol CYP/min)	
	5 μM	50 μM	5 μM	50 μM
CYP1A1	N.D.	N.D.	N.D.	N.D.
CYP1A2	N.D.	N.D.	N.D.	N.D.
CYP2A6	N.D.	N.D.	N.D.	N.D.
CYP2B6	N.D.	N.D.	N.D.	N.D.
CYP2C8	N.D.	N.D.	N.D.	N.D.
CYP2C9	N.D.	N.D.	N.D.	N.D.
CYP2C19	N.D.	N.D.	N.D.	N.D.
CYP2D6	N.D.	N.D.	N.D.	N.D.
CYP2E1	N.D.	N.D.	N.D.	N.D.
CYP2J2	N.D.	N.D.	N.D.	N.D.
CYP3A4	0.21 ± 0.002	0.63 ± 0.119	0.16 ± 0.005	0.57 ± 0.113
CYP3A5	N.D.	0.02 ± 0.002	0.01 ± 0.000	0.04 ± 0.003

N.D., not detected (<0.006 pmol/pmol CYP/min).

**Figure 3:**
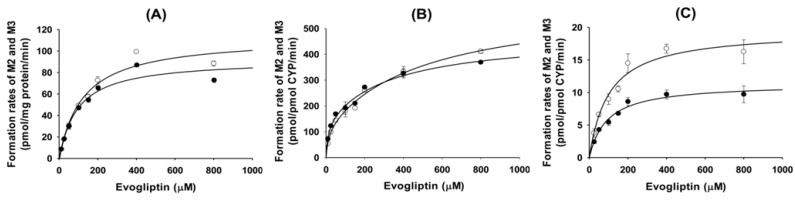
Enzyme kinetics for the formation of 4(*S*)-hydroxyevogliptin (M2, ●) and 4(*R*)-hydroxyevogliptin (M3, ○) from evogliptin in (**A**) human liver microsomes; (**B**) human cDNA-expressed CYP3A4; and (**C**) by human cDNA-expressed CYP3A5.

The enzyme kinetic profiles for the formation of major metabolites, 4(*S*)-hydroxyevogliptin (M2) and 4(*R*)-hydroxyevogliptin (M3) from evogliptin in pooled human liver microsomes followed single-enzyme kinetics (Figure 3A). The *K*_m_, *V*_max_, and *Cl*_int_ values of 4(*S*)-hydroxyevogliptin (M2) formation were 93.4 μM, 91.9 pmol/mg protein/min, and 1.0 μL/min/mg protein, respectively, and those of 4(*R*)-hydroxyevogliptin (M3) formation were 124.4 μM, 113.0 pmol/mg protein/min, and 0.9 μL∙min/mg protein, respectively (Table 3).

**Table 3:** Enzyme kinetic parameters for the metabolism of evogliptin to 4(*S*)-hydroxyevogliptin (M2) and 4(*R*)-hydroxyevogliptin (M3) in human liver microsomes and human cDNA-expressed CYP enzymes.

Human CYPs	*K*_m_ (μM)	*V*_max_	*Cl*_int_	*K*_m2_ (μM)	*V*_max2_	*Cl*_int2_
Formation of 4(*S*)-hydroxyevogliptin (M2)						
CYP3A4	10.9	128.8	11.8	330.1	340.7	1.0
CYP3A5	91.3	11.3	0.1	-	-	-
Liver microsomes	93.4	91.9	1.0	-	-	-
Formation of 4(*R*)-hydroxyevogliptin (M3)						
CYP3A4	13.0	112.8	8.7	511.6	497	1.0
CYP3A5	101.6	19.5	0.2	-	-	-
Liver microsomes	124.4	113.0	0.9	-	-	-

*V*_max_: pmol/min/pmol CYP for CYP3A4 and CYP3A5, pmol/min/mg protein for liver microsomes; *Cl*_int_: μL/min/pmol CYP for CYP3A4 and CYP3A5, μL/min/mg protein for liver microsomes.

The formation of 4(*S*)-hydroxyevogliptin (M2) and 4(*R*)-hydroxyevogliptin (M3) from evogliptin followed the isoenzyme equation in human cDNA-expressed CYP3A4 enzyme (Figure 3B), but showed single-enzyme kinetics in human cDNA-expressed CYP3A5 enzyme (Figure 3C). The enzyme kinetic parameters for the formation of 4(*S*)-hydroxyevogliptin (M2) and 4(*R*)-hydroxyevogliptin (M3) from evogliptin by the CYP3A4 and CYP3A5 enzymes indicate that CYP3A4 plays a prominent role in the formation of 4(*S*)-hydroxyevogliptin (M2) and 4(*R*)-hydroxyevogliptin (M3), with a minor contribution by CYP3A5 (Table 3).

**Figure 4:**
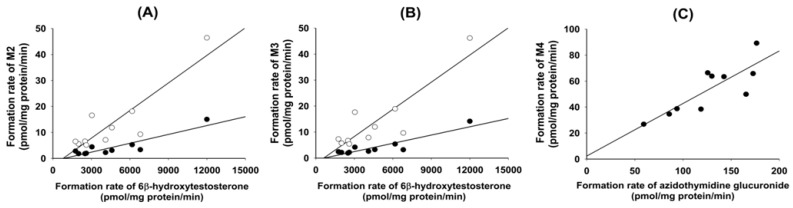
Correlation between the formation rates of (**A**) 4(*S*)-hydroxyevogliptin (M2) and (**B**) 4(*R*)-hydroxyevogliptin (M3) from 10 μM (●) and 50 μM (○) evogliptin and testosterone 6β-hydroxylase activity in 10 different human liver microsomes; (**C**) Correlation of the formation rates of 4(*S*)-hydroxyevogliptin glucuronide (M4) from 100 μM 4(*S*)-hydroxyevogliptin to azidothymidine glucuronidation activity in 10 different human liver microsomes.

The rates of formation of 4(*S*)-hydroxyevogliptin (M2) from 10 or 50 μM evogliptin in 10 different human liver microsomes were 1.8–15.0 pmol/mg protein/min and 5.2–46.4 pmol/mg protein/min, respectively (Figure 4A). The formation rates of 4(*R*)-hydroxyevogliptin (M3) from 10 or 50 μM evogliptin in 10 different human liver microsomes were 1.9–14.2 pmol/mg protein/min and 5.4–46.2 pmol/mg protein/min, respectively (Figure 4B). According to the correlation analysis using Pearson’s product–moment correlation coefficient, the formation rates of 4(*S*)-hydroxyevogliptin (M2) and 4(*R*)-hydroxyevogliptin (M3) in 10 different human liver microsomes were significantly correlated (*r*^2^ ≥ 0.882, *p <* 0.05) with testosterone 6β-hydroxylase activity, a marker enzyme of CYP3A4 (Figure 4A,B).

**Figure 5:**
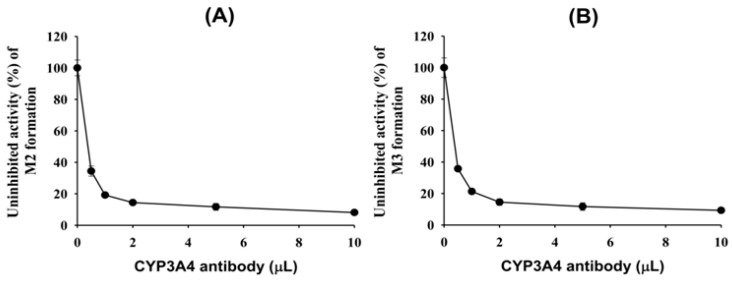
Effect of an anti-CYP3A4 antibody on the metabolism of evogliptin (50 μM) to (**A**) 4(*S*)-hydroxyevogliptin (M2) and (**B**) 4(*R*)-hydroxyevogliptin (M3) in pooled human liver microsomes.

To further characterize the CYP enzymes responsible for the formation of 4(*S*)-hydroxyevogliptin (M2) and 4(*R*)-hydroxyevogliptin (M3) from evogliptin, an immunoinhibition study was performed by pretreating pooled human liver microsomes with an anti-CYP3A4 antibody (Figure 5). The formation of 4(*S*)-hydroxyevogliptin (M2) and 4(*R*)-hydroxyevogliptin (M3) from evogliptin in pooled human liver microsomes was potently inhibited to a maximum of 90% by the CYP3A4 antibody, suggesting that CYP3A4 played the major role in the metabolism of evogliptin to 4(*S*)-hydroxyevogliptin (M2) and 4(*R*)-hydroxyevogliptin (M3) in human liver microsomes.

### 2.3. Characterization of Human UGT Enzymes Responsible for the Formation of 4-Hydroxyevogliptin Glucuronide (M4) from 4(S)-Hydroxyevogliptin

**Figure 6:**
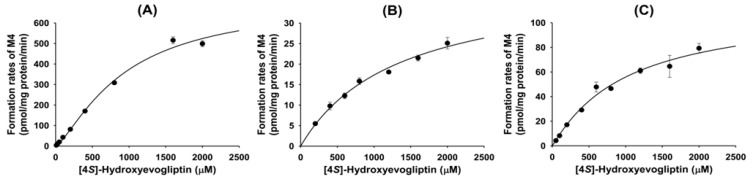
Enzyme kinetics for the metabolism of 4(*S*)-hydroxyevogliptin (M2) to 4(*S*)-hydroxyevogliptin glucuronide (M4) in (**A**) pooled human liver microsomes; (**B**) human cDNA-expressed UGT2B4; and (**C**) human cDNA-expressed UGT2B7.

The rates of formation of 4(*S*)-hydroxyevogliptin glucuronide (M4) from 4(*S*)-hydroxyevogliptin in pooled human liver microsomes showed a good fit to the Hill equations (Figure 6). The *K*_m_, *V*_max_, and *Cl*_int_ values for the formation of 4(*S*)-hydroxyevogliptin glucuronide (M4) were 927.9 μM, 711.4 pmol/mg protein/min, and 0.77 μL/min/mg protein, respectively.

Screening using human cDNA-expressed UGT 1A1, 1A3, 1A4, 1A6, 1A7, 1A8, 1A9, 1A10, 2B4, 2B7, 2B15 and 2B17 isoforms for the glucuronidation of 4(*S*)-hydroxyevogliptin (M2) to 4(*S*)-hydroxyevogliptin glucuronide (M4) showed that the UGT2B4 and UGT2B7 enzymes played major roles in the formation of M4, and that the UGT 1A1, 1A3, 1A4, 1A6, 1A7, 1A8, 1A9, 1A10, 2B15, and 2B17 enzymes did not contribute (Table 4).

**Table 4:** Formation rates of 4(*S*)-hydroxyevogliptin glucuronide (M4) from 50 and 300 μM 4(*S*)-hydroxyevogliptin (M2) in human cDNA-expressed UGTs (mean ± SD, *n* = 3).

Human UGTs	4(*S*)-Hydroxyevogliptin Glucuronide (M4) (pmol/mg protein/min)	
	50 μM	300 μM
UGT1A1	N.D.	N.D.
UGT1A3	N.D.	N.D.
UGT1A4	N.D.	N.D.
UGT1A6	N.D.	N.D.
UGT1A7	N.D.	N.D.
UGT1A8	N.D.	N.D.
UGT1A9	N.D.	N.D.
UGT1A10	N.D.	N.D.
UGT2B4	N.D.	6.22 ± 0.31
UGT2B7	7.46 ± 0.77	32.99 ± 1.04
UGT2B15	N.D.	N.D.
UGT2B17	N.D.	N.D.

N.D., not detected (<5 pmol/mg protein/min).

The rates of formation of 4(*S*)-hydroxyevogliptin glucuronide (M4) from 4(*S*)-hydroxyevogliptin (M2) followed single-enzyme kinetics with UGT2B4 and Hill equation kinetics with UGT2B7 (Figure 6). The *K*_m_, *V*_max_, and *Cl*_int_ values for the formation of 4(*S*)-hydroxyevogliptin glucuronide (M4) formation were 1,328 μM, 40.3 pmol/mg protein/min, and 0.03 μL/min/mg protein, respectively, for UGT2B4; and 1003.8 μM, 112 pmol/mg protein/min, and 0.1 μL/min/mg protein, respectively, for UGT2B7.

The rates of formation of 4(*S*)-hydroxyevogliptin glucuronide (M4) from 100 μM 4(*S*)-hydroxyevogliptin (M2) in 10 different human liver microsomes were 26.8–89.2 pmol/mg protein/min (Figure 4C). The correlation analysis demonstrated that the formation rates of 4(*S*)-hydroxyevogliptin glucuronide (M4) in 10 different human liver microsomes were significantly correlated (*r*^2^ = 0.834, *p <* 0.05) with UGT2B7-catalzyed azidothymidine *N*-glucuronidation (Figure 4C).

## 3. Discussion

*In vitro* metabolism of evogliptin using human hepatocytes, liver microsomes, and liver S9 fractions was investigated for the first time. Evogliptin and five of its metabolites, M1–M5, were characterized by LC-HRMS analysis following incubation with human hepatocytes (Figure 1A). Incubation of evogliptin with human liver S9 fractions in the presence of NADPH and PAPS resulted in the formation of 4(*S*)-hydroxyevogliptin (M2), 4(*R*)-hydroxyevogliptin (M3), and evogliptin *N*-sulfate (M5), as determined by LC-HRMS analysis (Figure 1B). Incubation of 4(*S*)-hydroxyevogliptin (M2) with pooled human liver microsomes in the presence of NADPH and UDPGA resulted in the formation of 4(*S*)-hydroxyevogliptin glucuronide (M4) and 4-oxoevogliptin (M1) (Figure 1C). Incubation of 4(*R*)-hydroxyevogliptin (M3) with pooled human liver microsomes in the presence of NADPH and UDPGA resulted in the formation of 4-oxoevogliptin (M1) (Figure 1D). Five metabolites were identified on the basis of the retention time and product ions by comparison with the corresponding authentic standards (Table 1, Figure 2). Evogliptin was metabolized to the following five metabolites in human liver: 4(*S*)-hydroxyevogliptin (M2) and 4(*R*)-hydroxyevogliptin (M3) via hydroxylation, evogliptin *N*-sulfate (M5) via sulfation, 4-oxoevogliptin (M1) from 4(*S*)-hydroxyevogliptin (M2) and 4(*R*)-hydroxyevogliptin (M3) via dehydrogenation, and 4(*S*)-hydroxyevogliptin glucuronide (M4) from 4(*S*)-hydroxyevogliptin (M2) via glucuronidation (Figure 3). Enzyme kinetic parameters for the formation of 4(*S*)-hydroxyevogliptin (M2) and 4(*R*)-hydroxyevogliptin (M3) from evogliptin in pooled human liver microsomes indicated that these compounds were the major metabolites.

To identify the CYP enzymes responsible for the formation of 4(*S*)-hydroxyevogliptin (M2) and 4(*R*)-hydroxyevogliptin (M3) from evogliptin, we performed a correlation analysis and immunoinhibition assay using human liver microsomes and performed a screening and evaluated enzyme kinetics using human cDNA-expressed CYP enzymes. Screening using cDNA-expressed human CYP enzymes showed that CYP3A4 played the predominant role in the formation of 4(*S*)-hydroxyevogliptin (M2) and 4(*R*)-hydroxyevogliptin (M3), with a minor contribution by CYP3A5 (Table 2). The formation rates of 4(*S*)-hydroxyevogliptin (M2) and 4(*R*)-hydroxyevogliptin (M3) from evogliptin in 10 different human liver microsomes were significantly correlated with CYP3A4-mediated testosterone 6β-hydroxylase activity (*r*^2^ ≥ 0.882, *p* < 0.05, Figure 4A,B). An anti-CYP3A4 antibody potently inhibited the formation of 4(*S*)-hydroxyevogliptin (M2) and 4(*R*)-hydroxyevogliptin (M3) by up to 90% (Figure 5) in pooled human liver microsomes. Therefore, the CYP3A4 enzyme played a major role in the hydroxylation of evogliptin to 4(*S*)-hydroxyevogliptin (M2) and 4(*R*)-hydroxyevogliptin (M3), with a minor contribution by CYP3A5, in human liver microsomes.

Metabolism of 4(*S*)-hydroxyevogliptin (M2) to 4(*S*)-hydroxyevogliptin glucuronide (M4) was mediated by human cDNA-expressed UGT2B4 and UGT2B7 (Table 4). These results were supported by a correlation analysis of the rates of formation of 4(*S*)-hydroxyevogliptin glucuronide (M4) in 10 different human liver microsomes and UGT2B7-catalzyed azidothymidine glucuronidase activity, which showed a significant correlation (*r*^2^ = 0.834, *p* < 0.05) (Figure 4C).

Formation of 4(*S*)-hydroxyevogliptin (M2) and 4(*R*)-hydroxyevogliptin (M3) from evogliptin was the major metabolic pathway catalyzed by CYP3A4/5. 4(*S*)-Hydroxyevogliptin (M2) was further metabolized to 4(*S*)-hydroxyevogliptin glucuronide (M4) by UGT2B4 and UGT2B7. 4(*S*)-hydroxyevogliptin (M2) and 4(*R*)-hydroxyevogliptin (M3) were further metabolized to 4-oxoevogliptin (M1). The CYP3A4 enzyme, which is responsible for the hydroxylation of evogliptin, is the most abundant CYP enzyme in the human small intestine and liver [12,16,17,18]. CYP3A plays a key role in the metabolism of ~30% of all drugs [12], and its activity shows wide interindividual variation due to genetic polymorphisms [16,17,18]. Genetic polymorphisms have also been described for UGT2B4 and UGT2B7, which were responsible for the glucuronidation of 4(*S*)-hydroxyevogliptin (M2) to M4 [19,20]. Therefore, the interindividual differences in the metabolism of evogliptin in humans is likely due to genetic variants of the CYP3A4, UGT2B4, and UGT2B7 enzymes responsible for the metabolism of evogliptin.

## 4. Experimental Section

### 4.1. Materials

Evogliptin, 4-oxoevogliptin, 4(*S*)-hydroxyevogliptin, 4(*R*)-hydroxyevogliptin, 4(*S*)-hydroxy- evogliptin glucuronide, and evogliptin *N*-sulfate were provided by Dong-A ST Co. (Yongin, Korea). William’s E medium, potassium phosphate monobasic, potassium phosphate dibasic trihydrate, reduced β-nicotinamide adenine dinucleotide phosphate tetrasodium salt (NADPH), Trizma^®^ Base, Trizma^®^ HCl, uridine-5-diphosphoglucuronic acid trisodium salt (UDPGA), alamethicin (from *Trichoderma viride*), 3′-phosphoadenosine-5′-phosphosulfate (PAPS), formic acid, and 1-methyl-4-phenylpyridinium iodide (MPPI, used as an internal standard) were purchased from Sigma-Aldrich Co. (St. Louis, MO, USA). Acetonitrile and water (LC-MS grade) were obtained from Fisher Scientific (Fairlawn, NJ, USA). The other reagents were of the highest quality available.

Cryopreserved human hepatocytes (catalog no. 454504), cryohepatocyte purification kit (catalog no. 454534), pooled and individual human liver microsomes (coded HG3, HH18, HK23, HG32, HK37, HG43, HH47, HG56, HG64, and HG74), human cDNA-expressed CYP enzymes (CYPs 1A1, 1A2, 2A6, 2B6, 2C8, 2C9, 2C19, 2D6, 2E1, 2J2, 3A4, and 3A5), human cDNA-expressed UGT enzymes (UGTs 1A1, 1A3, 1A4, 1A6, 1A7, 1A8, 1A9, 1A10, 2B4, 2B7, 2B15, and 2B17), and human-specific antibody for the immunoinhibition of human CYP3A4 (anti-CYP3A4) were purchased from Corning Life Sciences (Woburn, MA, USA).

### 4.2. Identification of the Metabolites of Evogliptin in Human Hepatocytes

Cryopreserved human hepatocytes were purified and recovered using a cryohepatocyte purification kit (Woburn) according to the manufacturer’s protocol. Purified human hepatocytes were resuspended in William’s E medium to a final density of 1.28 × 10^6^ cells/mL, and then 62.5 μL of human hepatocyte suspensions (8 × 10^4^ cells) and 62.5 μL of 100 μM evogliptin in William’s E medium were added to a 96-well plate and incubated for 4 h at 37 °C in a CO_2_ incubator. The reaction was terminated by the addition of 125 μL of ice-cold acetonitrile to each sample well, followed by centrifugation at 13,000 rpm for 4 min at 4 °C. Then, 40 μL of the supernatant were diluted with 60 μL of deionized water and an aliquot (5 μL) was injected onto LC-HRMS system to identify metabolites of evogliptin.

### 4.3. Identification of Metabolites of Evogliptin in Human Liver S9 Fractions

The incubation mixture consisted of 50 mM potassium phosphate buffer (pH 7.4), 10 mM magnesium chloride, pooled human liver S9 fractions (150 μg protein), 1 mM NADPH, 2 mM UDPGA, 0.2 mM PAPS and 10 μM evogliptin in a total volume of 300 μL. The incubation mixture was incubated for 60 min at 37 °C in a shaking water bath. The incubation was stopped by the addition of 300 μL of ice-cold acetonitrile, followed by centrifugation at 13,000 rpm for 4 min at 4 °C. The supernatant was evaporated using a vacuum concentrator and the residue was dissolved in 100 μL of 15% acetonitrile. An aliquot (5 μL) was injected onto the LC-HRMS system.

### 4.4. Identification of Metabolites of Evogliptin in Human Liver Microsomes

The incubation mixture consisted of 50 mM potassium phosphate buffer (pH 7.4), 10 mM magnesium chloride, pooled human liver microsomes (60 μg protein), 1 mM NADPH, 2 mM UDPGA, and 10 μM evogliptin in a total volume of 300 μL. The reaction mixture was incubated for 60 min at 37 °C in a shaking water bath. The incubation was stopped by the addition of 300 μL of ice-cold acetonitrile, followed by centrifugation at 13,000 rpm for 4 min at 4 °C, and the supernatant was evaporated. The residue was reconstituted in 100 μL of 15% acetonitrile, and an aliquot (5 μL) was analyzed by LC-HRMS.

### 4.5. Enzyme Kinetics of Evogliptin Metabolism to [4S]-hydroxyevogliptin (M2) and [4R]-hydroxyevogliptin (M3) in Human Liver Microsomes

Preliminary experiments demonstrated that the metabolism of evogliptin to 4(*S*)-hydroxyevogliptin (M2) and 4(*R*)-hydroxyevogliptin (M3) proceeded linearly with incubation time (10–30 min) and liver microsomal protein concentration (0.1–0.3 mg/mL). Thus, a 20 min incubation time and 0.15 mg/mL microsomal protein concentration were used in subsequent experiments.

The reaction mixture comprised 50 mM potassium phosphate buffer (pH 7.4), 10 mM magnesium chloride, pooled human liver microsomes (15 μg protein), and various concentrations of evogliptin (10 to 800 μM; final acetonitrile concentration not exceeding 0.5%, *v*/*v*) was preincubated for 3 min at 37 °C. The reaction was initiated by adding NADPH, and the mixture was further incubated (final volume of 100 μL) for 20 min at 37 °C in a shaking water bath. The reaction was terminated by adding 100 μL of MPPI (internal standard, 50 ng/mL) in ice-cold acetonitrile. The mixture was centrifuged at 13,000 rpm for 4 min at 4 °C. Subsequently, 50 μL of the supernatant were diluted with 50 μL of deionized water and an aliquot (5 μL) was injected onto the LC-MS/MS system.

### 4.6. Metabolism of Evogliptin in Human cDNA-Expressed CYP Enzymes

The reaction mixture comprised 50 mM potassium phosphate buffer (pH 7.4), 10 mM magnesium chloride, evogliptin (5 or 50 μM), and 12 human cDNA-expressed CYP enzymes (CYPs 1A1, 1A2, 2A6, 2B6, 2C8, 2C9, 2C19, 2D6, 2E1, 2J2, 3A4, and 3A5; 4 pmol) and was preincubated for 3 min at 37 °C. The reaction was initiated by addition of NADPH, and the mixture was further incubated (final volume of 100 μL) for 20 min at 37 °C in a shaking water bath. The reaction was terminated by adding 100 μL of MPPI (internal standard, 50 ng/mL) in ice-cold acetonitrile. The mixture was centrifuged at 13,000 rpm for 4 min at 4 °C. Continuously, 50 μL of the supernatant were diluted with 50 μL of deionized water and an aliquot (5 μL) was analyzed using the LC-MS/MS system.

For the enzyme kinetic experiments, various concentrations of evogliptin (10 to 800 μM; final acetonitrile concentration not exceeding 0.5%, *v*/*v*) were incubated with human cDNA-expressed CYP enzymes (CYPs 3A4 and 3A5; 4 pmol), 1 mM NADPH, and 10 mM MgCl_2_ in 50 mM potassium phosphate buffer (pH 7.4) for 20 min at 37 °C in a shaking water bath. After addition of ice-cold acetonitrile containing internal standard (MPPI, 50 ng/mL), the mixture was centrifuged and diluted as described above, and an aliquot (5 μL) was injected onto the LC-MS/MS system.

### 4.7. Correlation Analysis of Evogliptin Metabolism with Probe Substrate Activities in Human Liver Microsomes

Evogliptin (10 and 50 μM) was incubated with 10 different human liver microsomes (15 μg protein), 1 mM NADPH, and 10 mM magnesium chloride in 50 mM potassium phosphate buffer (pH 7.4) for 20 min at 37 °C in a shaking water bath. The correlation coefficients between the formation rates of 4(*S*)-hydroxyevogliptin (M2) or 4(*R*)-hydroxyevogliptin (M3) from evogliptin and specific CYP activities in human liver microsomes provided by Corning Life Sciences were evaluated by the Pearson product–moment correlation coefficient using Sigma Stat software (ver. 2.0; Systat Software Inc., San Jose, CA, USA). A *p* value < 0.05 was considered to indicate significance.

### 4.8. Immunoinhibition of Evogliptin Metabolism with an Anti-CYP3A4 Antibody

Immunoinhibition experiments were conducted by incubating pooled human liver microsomes with various amounts of an anti-CYP3A4 antibody for 15 min on ice, and then the reaction was initiated by the addition of 50 mM potassium phosphate buffer (pH 7.4), 50 μM evogliptin, 10 mM magnesium chloride, and 1 mM NADPH. Control incubations were performed using liver microsomes and 25 mM Tris buffer but without the anti-CYP3A4 antibody, which was prepared in this buffer.

### 4.9. Metabolism of 4(S)-Hydroxyevogliptin (M2) and 4(R)-Hydroxyevogliptin (M3) to 4-Oxoevogliptin (M1) and 4(S)-hydroxyevogliptin Glucuronide (M4) in Human Liver Microsomes

The incubation mixture consisted of 50 mM potassium phosphate buffer (pH 7.4), 10 mM magnesium chloride, pooled human liver microsomes (60 μg protein), 1 mM NADPH, 2 mM UDPGA, and 10 μM 4(*S*)-hydroxyevogliptin (M2) or 4(*R*)-hydroxyevogliptin (M3) in a total volume of 300 μL. The incubation mixture was incubated for 60 min at 37 °C in a shaking water bath. The incubation was stopped by the addition of 300 μL of ice-cold acetonitrile followed by centrifugation at 13,000 rpm for 4 min at 4 °C. Subsequently, the supernatant was evaporated and the residue was reconstituted in 100 μL of 15% acetonitrile. An aliquot (5 μL) was injected onto the LC-HRMS system.

### 4.10. Metabolism of 4(S)-Hydroxyevogliptin to 4(S)-Hydroxyevogliptin Glucuronide (M4) by Human cDNA-Expressed UGT Enzymes

To investigate the UGT enzymes involved in the glucuronidation of 4(*S*)-hydroxyevogliptin (M2), the incubation mixture (final volume of 100 μL) containing 50 mM Tris buffer (pH 7.4), 10 mM magnesium chloride, 0.025 mg/mL alamethicin, 4(*S*)-hydroxyevogliptin (M2) (50 or 300 μM), and 12 human cDNA-expressed UGT enzymes (UGTs 1A1, 1A3, 1A4, 1A6, 1A7, 1A8, 1A9, 1A10, 2B4, 2B7, 2B15, and 2B17; 10 μg protein) was preincubated for 3 min at 37 °C. The reaction was initiated by adding UDPGA, and the mixture was further incubated (final volume of 100 μL) for 20 min at 37 °C in a shaking water bath. The reaction was terminated by adding 100 μL of MPPI (internal standard, 50 ng/mL) in ice-cold acetonitrile. The mixture was centrifuged at 13,000 rpm for 4 min at 4 °C, 50 μL of the supernatant were diluted with 50 μL of deionized water, and an aliquot (5 μL) was analyzed using the LC-MS/MS system.

### 4.11. Enzyme Kinetics for the Metabolism of 4(S)-Hydroxyevogliptin to 4(S)-Hydroxyevogliptin Glucuronide (M4) in Human Liver Microsomes and cDNA-Expressed UGT Enzymes

Preliminary experiments showed that the glucuronidation of 4(*S*)-hydroxyevogliptin to 4(*S*)-hydroxyevogliptin glucuronide (M4) was linear with incubation time over 30 min and human liver microsomal protein concentration (0.1–0.3 mg/mL). Therefore, a 20 min incubation time and 0.2 mg/mL microsomal protein concentration were selected for enzyme kinetics experiments. The reaction mixture comprised 50 mM Tris buffer (pH 7.4), 10 mM magnesium chloride, 0.025 mg/mL alamethicin, pooled human liver microsomes (20 μg protein), and various concentrations of 4(*S*)-hydroxyevogliptin (M2) (10 to 2,000 μM; final acetonitrile concentration not exceeding 0.5%, *v*/*v*), and was preincubated for 3 min at 37 °C. The reaction was initiated by adding UDPGA, and the mixture was further incubated (final volume of 100 μL) for 20 min at 37 °C in a shaking water bath. The reaction was stopped by adding 100 μL of MPPI (internal standard, 50 ng/mL) in ice-cold acetonitrile. The mixture was centrifuged at 13,000 rpm for 4 min at 4 °C. Next, 50 μL of the supernatant were diluted with 50 μL of deionized water and an aliquot (5 μL) was injected onto the LC-MS/MS system.

For the enzyme kinetic study, various concentrations of 4(*S*)-hydroxyevogliptin (M2) (25 to 2000 μM; final acetonitrile concentration not exceeding 0.5%, *v*/*v*) were incubated with human cDNA-expressed UGT2B4 or UGT2B7 enzymes (20 μg protein), 2 mM UDPGA, 0.025 mg/mL alamethicin, and 10 mM magnesium chloride in 50 mM Tris buffer (pH 7.4) for 20 min at 37 °C in a shaking water bath. After adding ice-cold acetonitrile containing internal standard (MPPI, 50 ng/mL), the mixture was centrifuged and diluted as described above, and an aliquot (5 μL) was analyzed using the LC-MS/MS system.

### 4.12. Correlation Analysis of 4(S)-Hydroxyevogliptin Metabolism to 4(S)-Hydroxyevogliptin Glucuronide (M4) with Probe Substrate Activities in Human Liver Microsomes

The formation rates of 4(*S*)-hydroxyevogliptin glucuronide (M4) from 4(*S*)-hydroxyevogliptin (M2) were evaluated by incubating 4(*S*)-hydroxyevogliptin (100 μM) with 10 different human liver microsomes (20 μg protein), 2 mM UDPGA, 0.025 mg/mL alamethicin, and 10 mM magnesium chloride in 50 mM Tris buffer (pH 7.4) for 20 min at 37 °C in a shaking water bath. The correlation coefficients between the formation rates of 4(*S*)-hydroxyevogliptin glucuronide (M4) and specific UGT activities in human liver microsomes reported by Corning Life Sciences were determined by the Pearson product-moment correlation coefficient using Sigma Stat Software (Systat Software Inc.). A *p* value < 0.05 was considered to indicate significance.

### 4.13. LC-HRMS and LC-MS/MS Analysis of Evogliptin and Its Metabolites

To identify evogliptin and its metabolites, an Exactive Orbitrap mass spectrometer (Thermo Scientific, San Jose, CA, USA) coupled to an Accela ultra-performance liquid chromatography system was used. The separation was performed on a Unison-C8 column (3.0 μm, 2.0 mm i.d. × 75 mm; Imtakt Corporation, Kyoto, Japan) using a gradient elution of 5% acetonitrile in 0.1% formic acid (mobile phase A) and 95% acetonitrile in 0.1% formic acid (mobile phase B) at a flow rate of 0.3 mL/min: 14% B for 8.5 min, 14% to 90% B for 3.0 min, 90% B for 3.0 min, 90% to 14% B for 0.1 min, and 14% B for 5.5 min. The column and autosampler temperatures were 40 °C and 6 °C, respectively. The electrospray ionization (ESI) in positive and negative mode was used with the following electrospray source settings: spray voltage, 4.0 kV in positive mode and −3.0 kV in negative mode; vaporizer temperature, 350 °C; capillary temperature, 330 °C; sheath gas pressure, 35 arbitrary units; and auxiliary gas pressure, 15 arbitrary units. Higher-energy collision dissociation (HCD) with a collision energy of 10 to 40 eV was employed to investigate the fragmentation pattern of evogliptin and its metabolites. The mass measurement accuracy for evogliptin and its metabolites did not exceed 5 ppm, representing a good correlation between the theoretical mass based on the molecular elemental composition and the experimental mass obtained from the full-scan HRMS analysis. The proposed structures for the product ions of evogliptin and its metabolites were determined using the Mass Frontier software (ver. 6.0; HighChem Ltd., Bratislava, Slovakia).

For the quantification of evogliptin and its metabolites, a Nanospace SI-2 LC system (Shiseido, Tokyo, Japan) coupled to a tandem quadrupole mass spectrometer (TSQ Quantum Access; Thermo Fisher Scientific) was used. The ESI source settings for the ionization of evogliptin and its metabolites were as follows: spray voltage, 4.5 kV; vaporizer temperature, 350 °C; capillary temperature, 330 °C; sheath gas pressure, 35 arbitrary units; and auxiliary gas pressure, 15 arbitrary units. The collision energy for evogliptin and its five metabolites, M1–M5, were 18V, 12V, 13V, 13V, 18V, and 31V, respectively. To quantify evogliptin and its metabolites, selective reaction monitoring mode using specific precursor and product ion transitions was applied: *m*/*z* 402.2→346.1 for evogliptin, *m*/*z* 416.1→360.0 for 4-oxoevogliptin (M1), *m*/*z* 418.0→362.0 for 4(*S*)- and 4(*R*)-hydroxyevogliptin (M2 and M3, respectively), *m*/*z* 594.2→538.1 for 4(*S*)-hydroxyevogliptin glucuronide (M4), *m*/*z* 480.3→251.8 for evogliptin *N*-sulfate (M5), and *m*/*z* 170.1→128.2 for MPP^+^ iodide (internal standard). Peak areas for internal standard, evogliptin, and its metabolites were integrated using the Xcalibur^®^ software (Thermo Fisher Scientific).

### 4.14. Data Analysis

Results represent the average of three independent experiments using human liver microsomes and human cDNA-expressed CYP and UGT enzymes. The kinetic parameters (*K*_m_ and *V*_max_) for hydroxylation and glucuronidation were determined using the Michaelis-Menten equation [*V* = *V*_max_·S/(*K*_m_ + S)], the Hill equation [*V* = *V*_max_·S^n^/(*K*_m_^n^ + S^n^)], or the isoenzyme equation [*V* = *V*_max1_·(S/*K*_m1_)/(1 + S/*K*_m1_) + *V*_max2_·(S/*K*_m2_)/(1 + S/*K*_m2_)] with the Enzyme Kinetics software (version 1.3; Systat Software Inc.). In the above-mentioned equations, *V* was the velocity of the reaction at substrate concentration [S], *V*_max_ was the maximum velocity, *K*_m_ was the substrate concentration at which the *V* was half of *V*_max_, and *n* is the Hill constant. The intrinsic clearance (*Cl*_int_) was calculated as *V*_max_/*K*_m_.

## 5. Conclusions

Evogliptin is metabolized to 4(*S*)-hydroxyevogliptin (M2), 4(*R*)-hydroxyevogliptin (M3), 4-oxoevogliptin (M1), 4(*S*)-hydroxyevogliptin glucuronide (M4), and evogliptin *N*-sulfate (M5) in human liver preparations such as hepatocytes, microsomes and S9 fractions (Scheme 1). CYP3A4 played a major role in evogliptin hydroxylation to 4(*S*)-hydroxyevogliptin (M2) and 4(*R*)-hydroxyevogliptin (M3), with a minor contribution by CYP3A5. UGT2B4 and UGT2B7 are responsible for the glucuronidation of 4(*S*)-hydroxyevogliptin (M2) to 4(*S*)-hydroxyevogliptin glucuronide (M4). These results indicate that evogliptin metabolism may cause the interindividual differences in the pharmacokinetics of evogliptin in humans.
